# Internal Iliac Artery Embolization within EVAR Procedure: Safety, Feasibility, and Outcome

**DOI:** 10.3390/jcm11247399

**Published:** 2022-12-14

**Authors:** Federico Fontana, Andrea Coppola, Lucrezia Ferrario, Giuseppe De Marchi, Edoardo Macchi, Giada Zorzetto, Marco Franchin, Gabriele Piffaretti, Matteo Tozzi, Giulio Carcano, Filippo Piacentino, Massimo Venturini

**Affiliations:** 1 Diagnostic and Interventional Radiology Unit, ASST dei Sette Laghi, Università degli Studi dell’Insubria, 21100 Varese, Italy; 2 Vascular Surgery Unit, ASST dei Sette Laghi, Università degli Studi dell’Insubria, 21100 Varese, Italy; 3 Emergency and Transplant Surgery Unit, ASST dei Sette Laghi, Università degli Studi dell’Insubria, 21100 Varese, Italy

**Keywords:** EVAR, internal iliac artery, embolization

## Abstract

Background: This study is focused on Internal Iliac Artery (IIA) embolization in patients undergoing Endovascular Aneurysm Repair (EVAR). Our aims were: to establish the feasibility of the procedure; to assess the presence of endoleak (EL) and increase in the size of the sac at follow-up; to define the need for reintervention; and to evaluate mortality rate. Methods: In this retrospective single-center study, EVAR-treated patients with an embolization of IIA were chosen. Coils and vascular plug were used as embolizing agents. Results: A total of 49 participants were enrolled in the study (48 men and one woman) with a median age of 76 ± 12 years. Patients had no early EL in 87.75% of cases, 8.16% had type 1a EL, 2.04% type 1b EL, and 2.04% type 2 EL, with a comprehensive technical success of 95.91%. In the follow-up, at 1 month 72.22% remained without EL, at 6 months 70.97%, and at 1 year 81.48%. In the same period, the trend of type 1 EL was 5.56% (1 month), 3.23% (6 months), and 0% (1 year). For EL type 2: 22.22% at 1 month, 25.81% at 6 months, and 16.7% at 1 year. The overall mortality was 35.58% and the re-intervention rate was 16.33%. Conclusions: IIA embolization is a feasible and safe procedure. The presence of EL is not superior to EVAR procedures that do not involve embolization.

## 1. Introduction

From the moment J.C. Parodi conceived EVAR in 1991 [1] to now, this mini-invasive technique has been extensively used in the management of abdominal aortic aneurysms (AAA). Over time, indications have progressively extended and, sometimes, when the aneurysm involves the iliac axis, the exclusion of the internal iliac artery (IIA) is necessary to grant a proper landing zone [2].

The involvement of the common iliac arteries (CIA) occurs in about 20% of cases [3]. In those cases where the distal diameter of the common iliac artery is inadequate for the proper sealing of the prosthetic leg, the landing zone can be extended into the external iliac artery (EIA), and the hypogastric artery can be embolized to prevent the occurrence of a type 2 endo-leak (EL) [4]. Usually, hypogastric-hypogastric anastomosis can revascularize the excluded vascular district preventing gluteal claudicatio.

Aside from embolization options, in selected patients, there is the possibility to save the IIA by placing a dedicated iliac branch graft; iliac branched devices usually extend the aortic endoprosthesis with a bifurcated graft landing to both EIA and IIA [5]. In literature, indications regarding the embolization of the IIA are not completely clear [6,7,8,9,10]; with this study, our aim is to define its feasibility, benefits over time, and possible risks in the form of mortality.

## 2. Materials and Methods

### 2.1. Study Design

This retrospective, monocentric, observational study was performed according to the Declaration of Helsinki. All participants signed an informed consent, including publication of anonymized data.

Data from endovascular closure of hypogastric artery procedures, which occurred from 23 October 2012 to 30 May 2022, were prospectively collected.

Inclusion criteria were: age ≥ 18 y.o.; EVAR procedure; the embolization of the hypogastric artery (both intraprocedural and secondary after EL finding); and AAA involving common iliac artery. Exclusion criteria were the following: a history of severe allergy to contrast media; the diagnosis of pseudo-aneurysm; an embolization of other vessels than IIA; and the absence of valid consent to participate to the study.

### 2.2. Variables

The outcome variables of the study were defined as following:

- ELp: the absence of IIA EL at the end of procedure;
- EL1: the absence of IIA EL at 1 month;
- EL2: the absence of IIA EL at 6 months;
- EL3: the absence of IIA EL at 1 year;
- EL4: the absence of IIA EL at more than 1 year;
- Mortality: any death related to IIA EL;
- Re-intervention: any re-intervention related to IIA EL.

The dependent variables considered were: age, sex, laterality of the embolization (mono or bilateral), presence of branch, type of device used for embolization, execution timing, presence of dyslipidemia, hypertension, diabetes, and smoking, International Normalized Ratio (INR) and platelets values, use of antiplatelets, use of blood thinners. Devices used for embolization were Detachable Concerto Medtronic Coils (Micro Therapeutics Inc., Irvine, CA, USA) and Abbott’s Amplatzer Vascular Plugs (AVP; Abbott’s Medical, Playmouth, MN, USA).

### 2.3. Embolization Technique

**Figure 1:**
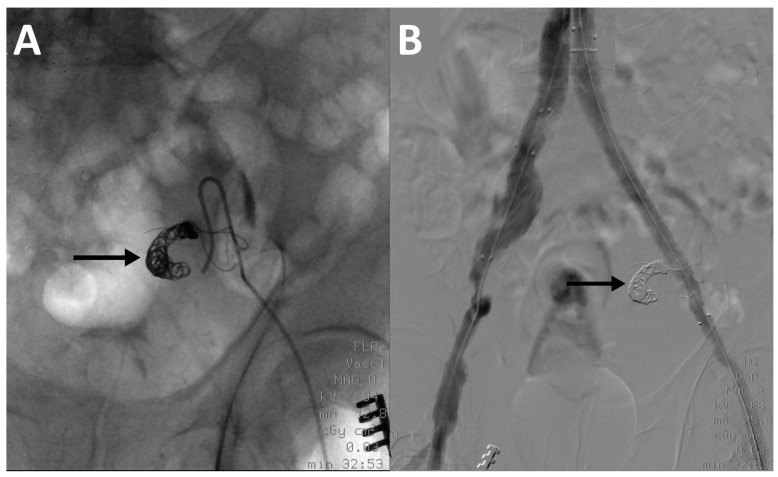
(**A**) Intraprocedural fluoroscopy image of a left ipogastrigc artery embolization with coils (arrows) using a Simmons-shape catheter. (**B**) Final digital subtraction angiography image at the end of the EVAR procedure showing complete exclusion of the left ipogastrigc artery.

Vascular access was gained via femoral artery. The hypogastric artery was catheterized using a Hook-shaped or a Simmons-shaped (Cordis Corporation, Miami Lakes, FL, USA) catheter. Angiography of the vessel was performed to assess the morphology of the artery and to identify the landing point for coils/vascular plug. For coils positioning, with coaxial technique, a micro-catheter (Terumo Progreat 2.7 Fr, Terumo Corporation, Shibuya, Tokyo, Japan) was moved forward and coils were released into the vessel. The micro-catheter was removed and another angiography was performed to check the correct position of coils. Embolizations performed with vascular plugs required the use of Simmons-shape catheter only and did not involve the use of a micro-catheter (Figure 1).

### 2.4. Follow-Up

**Figure 2:**
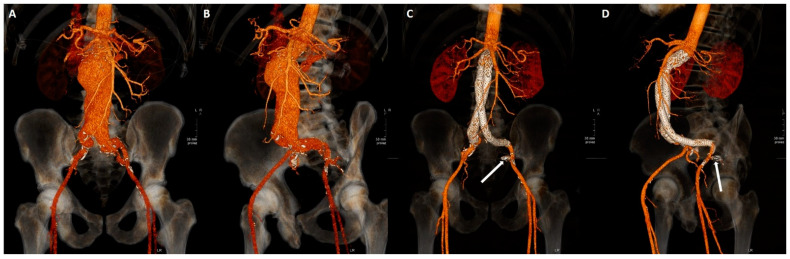
(**A**–**D**). Volume rendering reconstructions from CTA before (**A**,**B**) and after (**C**,**D**) EVAR procedure with left ipogastrigc embolization with coils (arrow).

Patients underwent clinical, contrast enhanced US (CEUS) and CT angiography (CTA) follow-up at demission time and at 1, 3, 6, 12 months from procedure, then annually. Clinical evaluation was performed at all checkpoints, CEUS and CTA were alternated starting with CEUS at 1 month. If CEUS was positive for EL, CTA was immediately performed (Figure 2).

### 2.5. Statistical Analysis

Data were anonymous and collected on an electronic dataset (Excel, Microsoft, Redmond, Washington, DC, USA). In all, 14 different variables were investigated as potential predictors of four different outcomes.

Descriptive statistics were produced for case demographics, clinical, and laboratory characteristics. Number and percentages were presented for categorical variables, mean and standard deviation (SD) were presented for continuous normally distributed variables, median and inter-quartile range (IQR) were presented for continuous non-normally distributed variables. Shapiro–Wilk test was used to assess normality.

According to data distribution, Student *t*-test and Mann-Whitney U-test were used to compare continuous variables. Crosstabs and Pearson’s Chi-square test (Fisher’s exact test where appropriate) were used to compare categorical variables.

SPSS version 25.0 (IBM, Armonk, New York, NY, USA) was used for all statistical analyses. In all cases, two-tailed tests were used. *p*-values were considered significant when <0.05.

## 3. Results

Forty-nine participants were enrolled in the study. There were 48 (97.96%) men and one (2.04%) woman, with a median age of 76 ± 12 (median ± IQR; range: 46–88) years. In all, 27 patients were treated with coils, 19 with AVP, and two with a combination of the two. Embolization was performed in 41 cases with vascular access from the same side as the IIA to be treated; in eight cases with the cross-over technique. Exhaustive descriptive statistics for case demographics, clinical, and laboratory characteristics are presented in Table 1. All continuous variables presented non-normal distribution according to Shapiro–Wilk test.

Overall, 43/49 (87.75%) patients underwent embolization of IIA without showing EL in fluoroscopy at the end of the procedure. Only 6/49 (12.25%) of patients had a precocious EL (within one month): there were 4 type Ia EL, 1 type Ib EL, and 1 type II EL; excluding type Ia EL, which cannot be caused by hypogastric artery, the comprehensive success rate was 95.91% (47/49). Regarding follow up, it is noticeable that 36/49 (73.47%) of the originally selected patients had first month follow up, 34/49 (69.39%) reached 6-months follow up and 27/49 (55.10%) the one-year follow up control appointment. At one month, 8/36 (22.22%) of the group had type 2 EL, at six months 8/34 (25.81%) and after one-year 5/27 (18.52%).

For what concerns the treatment of bilateral aneurysm of the IIAs, only three of our 49 (6.12%) patients underwent bilateral IIA exclusion. One of them is still alive, one died 48 months after the procedure, and one died after 10 months. Six out of 49 patients (12.24%) in whom one hypogastric artery was preserved and the contralateral was embolized, one died the day after the procedure while the other five are still alive. None of the deaths was related to a complication of the vascular procedure or the progression of the aneurysmatic disease.

**Figure 3:**
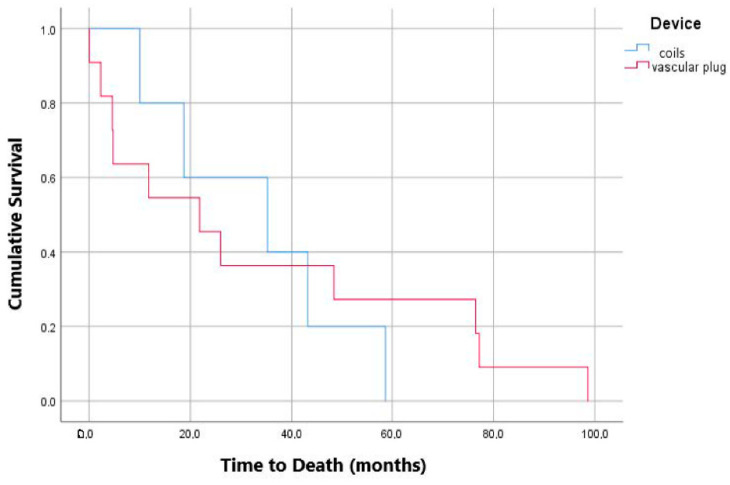
Kaplan-Meier curve showing relationship of mortality of patients treated with coils and vascular plug.

**Figure 4:**
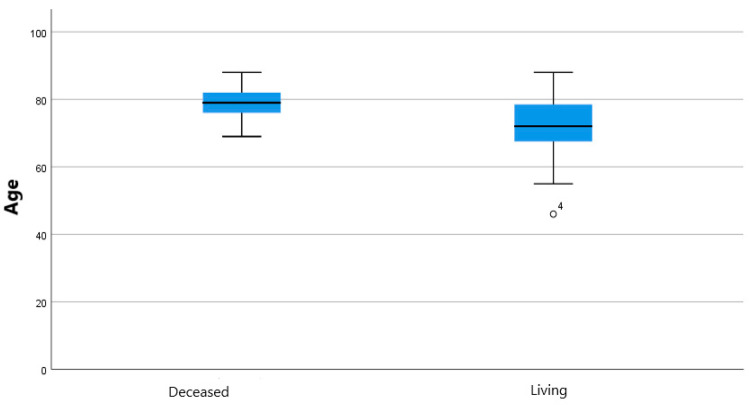
Boxplot showing relationship of mortality of patients by age. Outliers are marked with a circle and the case number.

**Figure 5:**
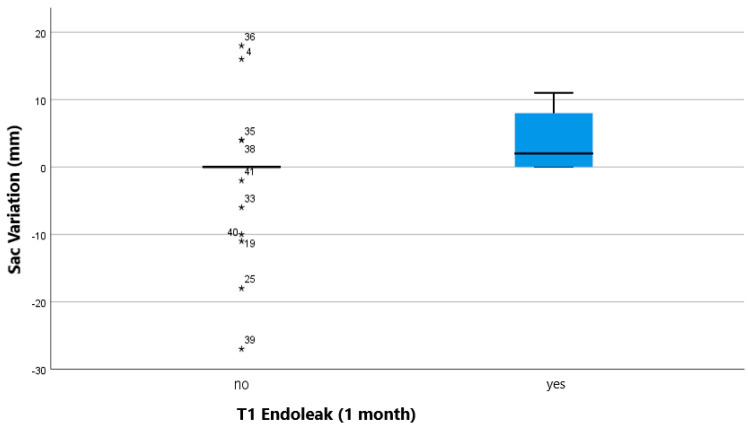
Boxplot showing relationship of endoleak occurrence at 1 month and aneurysmal sac variation at the same temporal endpoint. Outliers are marked with a “*” and the case number.

Crosstabs with Pearson’s Chi-square test (Fisher’s exact test where appropriate) and Mann-Whitney U test analysis for continuous variables showed statistically significant correlation between mortality and type of device (*p*: 0.01; odds ratio for coils against coils of 0.173; 95% confidence interval: 0.046–0.658; Figure 3), age and mortality (*p*: 0.016; Figure 4), sac variation (mm) and T1 EL (1 month; *p*: 0.014; Figure 5).

## 4. Discussion

Since J.C. Parodi introduced the EVAR technique [1], several limitations have emerged regarding the placement of the endoprosthesis. One of these limitations concerns the complex anatomies of the infrarenal abdominal aorta and particularly the common iliac artery aneurysm. Such aneurysm, on the one hand, makes it difficult to secure a landing zone, and on the other hand, it was almost a foregone conclusion that patients of this type would later show EL [9,10].

The main objective of this study was to determine the actual feasibility of embolization of the IIA and to consider the presence of EL as a yardstick. Different mechanical devices [2,11,12,13,14] or embolic agents [8,15,16] were previously described in IIAs embolization. Our results showed that 87.75% of patients could undergo the embolization of IIA without showing EL in fluoroscopy at the end of the procedure. Only 4.09% of patients had a precocious IIA EL, with a 95.91% comprehensive success rate. Similar technical success rates can be found in literature; Chun et al. [17] showed a success rate of 95.7% in a sample twice the size of this one. Coils and plugs were preferred as embolizing agents. This is reflected in similar studies, such as the Chun et al. and Kotoku et al. studies [17,18].

We analyzed the patient that presented type 2 EL at the end of the procedure. It was a 76 years old male who presented a hypogastric artery aneurysm, which complicated the anatomical situation. The case was treated with AVP for this reason, but presented a feeble type 2 EL at the end of the procedure. It was treated conservatively and it disappeared before the one-month CTA follow-up. This kind of technical failure is described in the literature. Incomplete exclusion of the IIA is in fact the most common reason for technical failure, even in the George et al. systematic review regarding isolated IIA aneurysms [19].

Even if we had no EL when the IIA was embolized after the bifurcation, we consider proximal embolization of the IIA relevant for a better technical result. Bosanquet et al. [2], in their systematic review, pointed out how this technique can grant a better clinical outcome for patients.

Regarding our secondary end point, there has only been one case of EL that could be due to the previously embolized IIA, i.e., a type 1b. Nevertheless, we decided to keep the data collected in the follow-up and collaterally consider the results from a statistical point of view. As displayed in Table 1, one-month EL rate (27.78% total) is aligned with literature data, which depict for a EL rate between 15 and 30% in the same observational period [20,21]. However, the most interesting thing to note is presented in Figure 5; we see how sac growth is related to the presence of EL at one month (*p*: 0.014). These data agree with what can be found in literature, since Mulay et al. and Marrewijk et al. found a correlation between sac enlargement and EL [20,21].

With regards to the need for reintervention, 16.33% of our patients needed one (8/49), including several procedures such as the embolization of other vessels (inferior mesenteric artery, accessory renal artery, and celiac tripod), the percutaneous embolization of the sac, and open repair to substitute the endograft. Timaran et al. [22] had a better overall result (12 re-interventions on a sample of 348 patients), but the study considers patients treated simply with EVAR, who did not undergo an embolization of the hypogastric artery.

In a study that comes closest to the characteristics of ours, by Wang et al. [23], the overall re-intervention rate stands at 7.7%. Such a low rate could also be related to the smaller sample size considered (26 patients), and also the fact that a bifurcated endoprosthesis was not always necessary.

Embolization is certainly not a risk-free procedure. A systematic review of the literature by Kontopodis et al. [24] points out how the simple coverage of the IIA may lead to fewer major complications than a preventive embolization, while the re-intervention rate is similar in the two groups, even if limited data has been analyzed. What emerges from a large retrospective study by Papazoglou et al. [25] is that the incidence of buttock claudication is not different in the group treated with coil and the group that received only IIA coverage; in this second group 6% developed type 2 ELs, even if none of them required re-intervention. Bosanquet et al. [2], in their review, found that type 2 ELs occurred more in covered IIAs than in occluded IIAs. In general, the literature is not unanimous on the subject, partly because studies are often carried out on small cohorts of patients. In our study, we chose not to focus on the individual complications of this procedure but on the general mortality rate and the need to re-intervene, both surgically and endovascularly, on the aneurysmal pathology. Our findings on this subject were that, of 48 patients for whom we could find data about mortality, 64.58% were still living in a period of observation that goes from the day after the procedure up to 109 months, meaning a mortality rate 15% higher than the EVAR trial 1 [26]. The most plausible explanation for this difference is that patients with greater impairment of the vascular system also present greater general clinical impairment. In the Chen et al. [27] study, late mortality rate was 17% (four patients out of 23 in a range of 38 to 72 months), a percentage smaller than ours, but distributed over a shorter time span. In addition to this, we noticed an age threshold at the time of the procedure beyond which death is more probable, as shown in Figure 4; patients over 79 years have less chance of survival than younger patients (*p*: 0.016). Speaking of mortality, another interesting relationship is to note is the one that involves the embolization material. In our findings, people treated with coils have an overall higher survival rate than people treated with plugs (OR: 1.533); this finding seems in contrast with the Bosanquet et al. [2] review, reporting a higher rate of complications related with coil embolization. Since we considered mortality as a whole, the two phenomena (most frequent complications and mortality) may not be closely related. On the other hand, looking at Figure 3, we can see how coils lead to death in less time than vascular plugs (60 months compared to 99 months). Our first explanation to this phenomenon is that plugs were more widely used before 2015 in our center, but among the five patients treated with coils in the very same period (from 11 June 2012 to 17 November 2015), three are still living, one survived 62 months after the procedure, and one survived 17 months.

Since literature reports increased the rate of complications in excluding both IIAs [2], we observed mortality in our patients who may fall into this subgroup: despite our small sample, the data speak in our case in favor of saving one of the two arteries; this matches European and American Guidelines that recommend the revascularization of at least one of the IIAs in iliac aneurysms involving both the iliac arteries [28,29].

The present study has some limitations: it is a retrospective study; the size of the sample is not so conspicuous; the distribution between two sexes is poor; and some controls carried out in other hospitals escape follow-up. Beside these limits, the study is expandable by comparison with branches.

## 5. Conclusions

In this retrospective analysis, with the widespread use of EVAR, this work suggests the feasibility of it even in patients with iliac artery aneurysm, thanks to the embolization of the IIA that can grant a better landing zone and showed a low prevalence of precocious EL. During the observational period, our patients showed an EL rate not so different from the EVAR procedure, that does not include IIA embolization. When deciding how to treat a patient with both iliac arteries aneurysmatic, to save at least one of the hypogastric arteries by branch positioning seems a better choice.

## Figures and Tables

**Table 1:** Descriptive statistics of the 49 patients enrolled in the study.

Variable	N	Value	Range	Variable	N	Value	Range
**Age**	49			**secondary EL (1 month)**	36		
median; IQR		76 (69.5–81.5)	46–88	*no*		26 (72.22%)	-
**Sex**	49			*type 1*		2 (5.56%)	
*M*		48 (97.96%)	-	*type 2*		8 (22.22%)	
*F*		1 (2.04%)		**T2 (6 months)**	34		
**Laterality**	49			*no*		22 (70.97%)	-
*monolateral*		46 (93.88%)	-	*type 1*		1 (3.23%)	
*bilateral*		3 (6.12%)		*type 2*		8 (25.81%)	
**Branch**	49			**T3 (1 year)**	27		
*Embolization only*		43 (87.76%)	-	*no*		22 (81.48%)	-
*Embolization + branch*		6 (12.24%)		*type 1*		0 (0%)	
**Device**	48			*type 2*		5 (18.52%)	
*coils*		27 (56.25%)		**T4 (>1 year)**	12		
*plug*		19 (39.58%)	-	*no*		9 (75%)	-
*Coils + plug*		2 (4.17%)		*type 1*		1 (8.3%)	
**Execution Timing**	49			*type 2*		2 (16.7%)	
*contestual*		46 (93.9%)	-	**How many years after**	12		
*later stage*		3 (6.1%)		*mean; SD*		4.5 ± 2.78	2–9
**Dyslipidemia**	49	31 (63.27%)	-	**Last control**	49		
**Hypertension**	49	37 (75.51%)	-	*mean; SD*		19.3 ± 26.1	0–108
**Diabetes Mellitus**	49	11 (22.45%)	-	**Type of control**	49	-	
**Smoking**	49	20 (40.82%)	-	*CEUS*		9 (18.37%)	-
**INR**	49			*TC*		24 (48.94%)	
median; IQR		1.08 (1–1.15)	0.92–3.25	*TC + CEUS*		10 (20.41%)	
**PLT** (10^9^/L)	49			**Sac Variation (mm)**	46		
median; IQR		178 (150.5–217.5)	61–425	median; IQR		0 (0–2)	−27–+18
**Antiplatelets**	49			**Mortality**	48		
*no*		12 (24.49%)		*deceased*		17 (35.42%)	-
*yes*		29 (59.18%)	-	*living*		31 (64.58%)	
*2*		8 (16.33%)		**Time to exitus (months)**	17		
**Oral Bloodthinners**	49	5 (10.2%)	-	*mean; SD*		32.55 ± 30.03	0.03–98.6
**early EL (technical sucess)**	49			**Reintervention**	49	8 (16.33%)	-
*no*		43 (87.75%)	-				
*type 1a*		4 (8.16%)					
*type 1b*		1 (2.04%)					
*type 2*		1 (2.04%)					

## Data Availability

The data presented in this study are available on request from the corresponding author. The data are not publicly available due to privacy restrictions.
